# 高低转移大细胞肺癌细胞株L9981和NL9980间甲基化差异基因的初步研究

**DOI:** 10.3779/j.issn.1009-3419.2010.08.02

**Published:** 2010-08-20

**Authors:** 慧 吕, 惠琴 闫, 岷 王, 永文 李, 海粟 万, 红雨 刘, 蘅 吴, 清华 周

**Affiliations:** 1 300052 天津，天津医科大学总医院，天津市肺癌研究所，天津市肺癌转移与肿瘤微环境重点实验室 Tianjin Key Labortory of Lung Cancer Metastasis and Tumor Microenviroment, Tianjin Lung Cancer Institute, Tianjin Medical University General Hospital, Tianjin 300052, China; 2 300052 天津，天津医科大学总医院胸部肿瘤中心肿瘤内科 Department of Medical Oncology, Tianjin Thoracic Cancer Center, Tianjin Medical University General Hospital, Tianjin 300052, China

**Keywords:** 甲基化基因芯片, DNA甲基化, 转移, 肺肿瘤, MeIP chip, DNA methylation, Metastasis, Lung neoplasms

## Abstract

**背景与目的:**

肺癌是人类肿瘤中最容易发生侵袭转移的恶性肿瘤之一。影响肺癌患者预后及生存的最主要因素是远处转移。本文旨在分析具有相似遗传背景和不同转移潜能的人大细胞肺癌细胞株间基因甲基化的差异，为深入研究肿瘤转移机制提供充分的目标基因选择和发现肿瘤相关的新基因的机会。

**方法:**

应用全基因组甲基化芯片杂交技术分析具有相同遗传背景和不同转移潜能的人大细胞肺癌细胞株L9981和NL9980之间差异甲基化基因，并运用生物信息学的方法分析这些差异基因。

**结果:**

与NL9980相比，在L9981细胞株中有1 552个高甲基化DNA片断共涉及735个基因，其中656个已知基因及79个未知基因。低甲基化DNA片段1 787个，涉及809个基因，其中698个已知基因及111个未知基因。这些基因涉及细胞生物进程及其调节、基因表达、信号传导、细胞通讯、细胞运动、细胞粘附及血管生成等。

**结论:**

抑癌基因及信号通路负向调节基因的高甲基化及癌基因、细胞粘附因子的低甲基化可能与L9981的侵袭性及克隆能力强有关。

## Abstract (en)

**Background and objective:**

Invasion and metastasis is one of malignant phenotype of lung cancer and the important cause of the death of lung cancer patients. The aim of this study is to explore the methylation profile difference in different metastatic potential cell lines.

**Methods:**

To compare the DNA methylation profile of two large cell lung cancer cell lines with different metastatic potential by MeIP chip hybridization, hypermethylated and hypomethylated genes of L9981 cell lines were analyzed online in NIH-DAVID, KEGG and MILANO webside.

**Results:**

Compared with NL9980 cell line, 735 genes are hypermethylated in L9981, including 656 known genes and 79 unknown genes; 809 gene are hypermethylated in L9981, including 698 known genes and 111 unknown genes; the different genes are involved in cell process, signal transduction, cell communication, cell adhesion, cell motility, and angiogenesis.

**Conclusion:**

Hypermethylation of suppressor genes and negative regulator of signal transduction and hopomethylation of oncogene and cell adhesion molecules (CAMs) in L9981 may promote metastasis of tumor cells.

肺癌是我国发病率和死亡率增长最快的恶性肿瘤之一，也是人类肿瘤中最容易发生侵袭转移的恶性肿瘤之一。影响肺癌患者预后和生存的最主要原因是肺癌的远处转移^[1]^。因此，研究和阐明调控肺癌侵袭转移的分子机制具有重要意义。

我们前期的研究^[2]^从人大细胞肺癌细胞株WCQH29801分离构建不同转移潜能的人大细胞肺癌细胞系NL9980和L9981。L9981细胞在体外具有较强的克隆形成能力和侵袭力，在裸鼠体内的自发性肺转移能力为100%，均显著高于NL9980细胞^[2-5]^。因此我们推测二者之间差异表达基因可能与L9981的侵袭及克隆能力强有关。表观遗传学是研究无DNA序列变化、可遗传的基因表达（活性）的改变的一门学科^[6]^。DNA甲基化是表观遗传的主要方式，它是由DNA甲基转移酶介导，在胞嘧啶的第5位碳原子上加上一甲基基团，使之变成5-甲基胞嘧啶（5 mC）的化学修饰过程，当基因启动子高甲基化时使基因失活，去（低）甲基化则使基因重新开放^[7]^。因此我们采用甲基化芯片杂交技术^[8, 9]^对这两个细胞株的差异甲基化基因进行比较，从而进一步探讨甲基化在肺癌转移中的作用。该技术主要是利用特定抗体对甲基化的胞嘧啶进行免疫沉淀反应（immunoprecipitation）使甲基化和非甲基化的胞嘧啶分离，并对分离出的甲基化DNA片段进行基因芯片杂交，实现高通量的检测。该方法对任意序列背景下的甲基化胞嘧啶均能实现免疫沉淀，分辨率相对较高，信息量较大，有助于发现两者的差异甲基化基因及新基因^[10]^。

## 1 材料与方法

### 1.1 细胞株

NL9980（低转移大细胞肺癌细胞株）、L9981（高转移大细胞肺癌细胞株）均由天津市肺癌研究所提供。

### 1.2 常用试剂及设备

精制小牛血清和RPMI-1640培养基均购自Gibco公司；使用120 mmol/L的NaOH溶液配成1 mmol/L的贮备液，4 ℃贮存；TIANamp Genomic DNA Kit血液/细胞/组织基因组DNA提取试剂盒（Tiangen, Cat. No. DP304-03）；Resi: MBD2b蛋白-sepharose-4B柱（上海生物芯片公司）；QIAquick PCR purification kit（Qiagen, Cat. No. 28106）；Cy3、Cy5 9mer Wobble（50, 200 O.D.）（TriLink Bio-technologies, Cat. No. N46-0010）Linker（15P）oligo JW102（40 μM）；oligo JW103（40 μM）；CPK6 48mer oligos（上海生工生物工程技术服务有限公司合成）；NimbleGen Hybridization Kit 40 Refill（Nimblegen, Cat. No. KIT005-2）；杂交炉（HB-1000 HYBRIDIZER）美国UVP LAB-ORATORY公司；扫描仪（AXON INSTRUMENTS GENE PIX 4000B）美国AXON公司；NimbleScan^TM^ 2.2：购自NimbleGen公司。

### 1.3 基因芯片

项目所用芯片SBC human CHIP：定制于上海生物芯片有限公司。探针为Nimblegen设计，设计原则是：一段DNA序列 > 250 bp，GC含量 > 57%时，认为这一段为CpG岛。探针一共有3 678 702条，每个岛所含的探针个数，从10多到30多不等，大小均为50 nt在芯片中平均分布。

### 1.4 提取L9981、NL9980细胞株的基因组DNA

用TIANamp Genomic DNA Kit提取基因组DNA，详细操作方法和原理见TIANamp Genomic DNA Kit protocol。并用分光光度计定量及普通凝胶电泳检测质量。

### 1.5 对L9981、NL9980细胞株的基因组DNA进行超声破碎

用超声破碎仪将L9981、NL9980细胞的基因组DNA破碎成DNA片段。并用分光光度计定量及普通凝胶电泳检测质量。

### 1.6 甲基化免疫沉淀方法富集甲基化DNA

通过Resi: MBD2b蛋白-sepharose-4B柱富集甲基化片断。然后用连接介导PCR（ligation-mediated PCR, LM-PCR）的方法^[11]^进行2次扩增。并用分光光度计定量及普通凝胶电泳检测质量。

### 1.7 芯片杂交及分析

应用寡核苷酸直接掺入法分别将Cy3和Cy5标记扩增的L9981及NL9980细胞株的DNA片段，然后NimbleGen Array Hybridization Kit与高通量甲基化芯片进行杂交产生2张芯片。使用NimbleScan^TM^ 2.2（NimbleGen）分析结果。用SignalMap^TM^对所得数据进行分析。对芯片进行标准化，将2张芯片的数据调至同一个水平。

### 1.8 生物信息学分析

用GenBank注释基因，并将甲基化差异基因分别上传至DAVID数据库（http://david.abcc.ncifcrf.gov/home.jsp）进行基因类型（GENE ONTOLOGY, GO）分类，KEGG数据库进行信号传导通路的分类，以及MILANO网站（http://milano.md.huji.ac.il）进行文献检索。

## 2 结果

### 2.1 L9981、NL9980细胞株的基因组DNA

两组细胞抽取提纯后，琼脂糖凝胶电泳结果显示，基因组DNA条带清晰完整，长度约20 kDa，无降解，无蛋白质杂带（图 1）。分光光度计结果显示L9981及NL9980 DNA的OD_260_/ OD_280_的比值均在1.7-2.0之间，提示所提的DNA纯度高。

**1:**
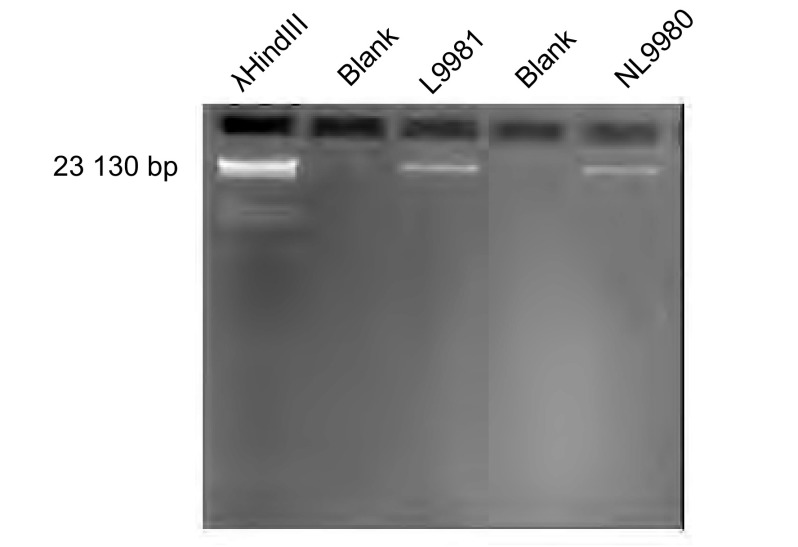
L9981和NL9980细胞株基因组DNA凝胶电泳图 Gel electrophoresis of genome DNA of L9981 and NL9980

### 2.2 超声破碎后的L9981、NL9980细胞株的基因组DNA片段

对基因组DNA进行超声破碎后行凝胶电泳检测，L9981及NL9980 DNA电泳条带呈弥散状，片段长度在500 bp-1 300 bp之间（图 2），当DNA片段长度在300 bp-1 300 bp之间时便于与Resi:MBD2b蛋白-sepharose-4B柱结合。

**2:**
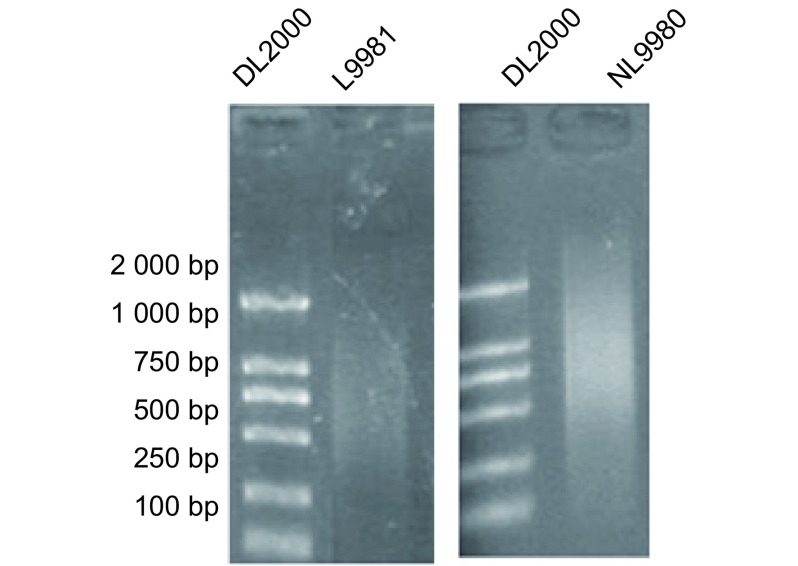
L9981和NL9980细胞基因组DNA超声破碎后凝胶电泳图 Gel electrophoresis of DNA frangments of L9981 and NL9980 by sonication

### 2.3 甲基化DNA

在普通凝胶电泳结果示L9981及NL9980 DNA电泳条带呈弥散条带，与基因破碎后的弥散条带大致相同，在300 bp-1 000 bp之间，在500 bp附近亮度最高（图 3）。

**3:**
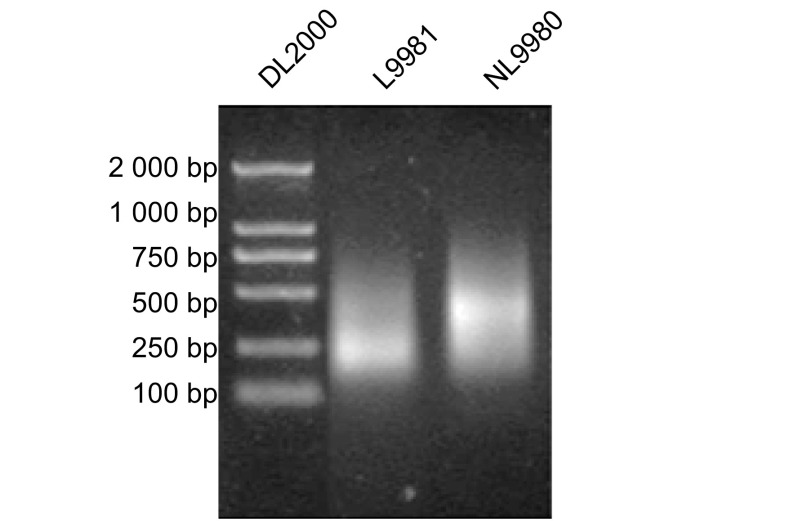
甲基化DNA的PCR扩增 PCR amplification of methylated DNA

### 2.4 芯片杂交及分析结果

图 4所示芯片完整，无高信号和低信号的团块和划痕，芯片的中央、四个角及中线与四个边交点处的红色十字架及平均分布的红点显示完全且清楚，说明芯片的杂交、洗涤、扫描步骤质量良好。Cy3通常显示绿色，Cy5显示红色，当某位点在芯片上呈绿色，则表示Cy3信号强，提示该位点在L9981中表现为高甲基化；反之，当位点呈红色，则表示Cy5信号强，提示该位点在L9981中表现为低甲基化；而位点呈黄色则表示该位点在两个细胞株内无甲基化差异。由图 4可见芯片整体呈黄色，意味着大部分的位点无差别，符合小部分位点甲基化程度变化的假设。

**4:**
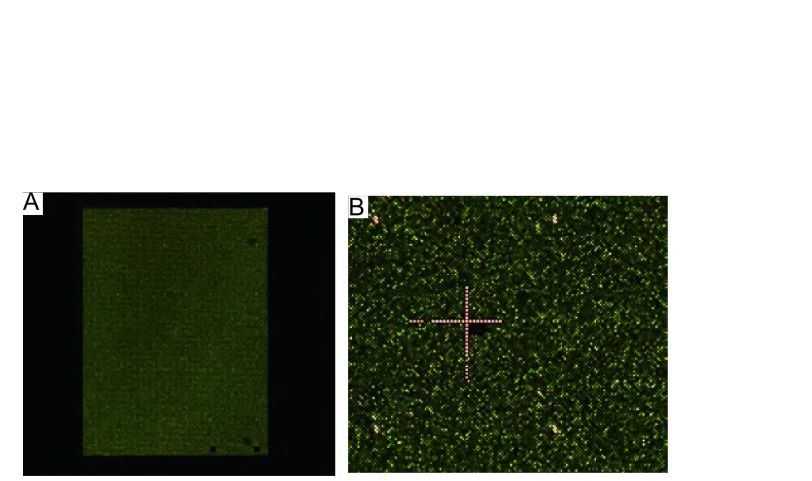
芯片图片。A：芯片扫描的全景图；B：质控部分的放大图，可以清晰看到中央十字及四角红点。 The picture of chip. A: The scanning picture of chip; B: The enlarged picture of quality parts, in which red cross in center and red pots in corner are clear.

图 5所示为标准化前后的M-A图，竖坐标0水平以上为上调基因，0水平以下为下调基因。根据文献^[12]^，我们取Cy3/Cy5 > 2，即竖坐标log2（Cy3/Cy5） > 1表示在L9981中高甲基化的基因；Cy3/Cy5 < 0.5，即竖坐标log2（Cy3/ Cy5） < -1表示在L9981中低甲基化的基因；而当Cy3/Cy5在0.5-2或log2（Cy3/Cy5）在-1–1之间时，表明该基因在L9981及NL9980中无甲基化差异。通过分析，芯片中共显示29 369个甲基化位点，其中19 369个为已知基因CpG岛。其中大部分基因的Cy3/Cy5比值在0.5-2之间，我们认为这些基因在L9981和NL9980之间无甲基化差异，这与芯片扫描图及M-A plot的结果一致。这些无甲基化差异的基因包括*nm23-H1*基因。只有少量的基因出现了甲基化的差异（表 1）。在L9981细胞株中有1 552个高甲基化DNA片断，涉及735个基因，其中包括656个已知基因及79个未知基因。低甲基化DNA片段1 787个，涉及809个基因，其中698个已知基因片段及111个未知基因。

**5:**
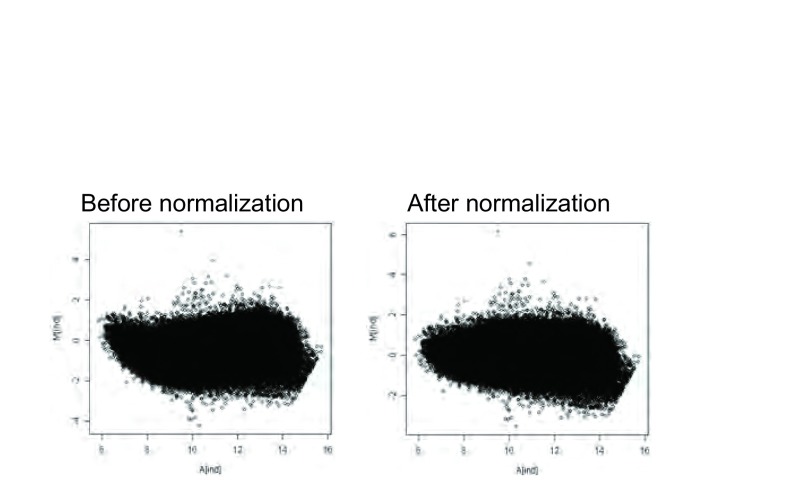
标准化前后的M-A图 The M-A plot before and after the normalization

**1:** L9981中高甲基化和低甲基化基因数目 The number of hypermethylated and hypomethylated genes in L9981

	Hypermethylation Cy3/cy5 > 2 (*n*)	Hypomethylation Cy3/cy5 < 0.5 (*n*)
Total	735	809
Known gene	656	698
Unknown gene	79	111

### 2.5 基因筛选结果

在这些差异基因中无*nm23*，表示*nm23*基因在这两个细胞株中无甲基化状态差异。通过GO分类我们发现：这些差异甲基化基因主要集中在细胞生物进程及其调节、代谢及其调节、基因表达、信号传导、细胞通讯、细胞运动、细胞粘附及血管生成等相关的基因，其中基因分类及基因数目的部分结果见表 2。通过MILANO对这些差异基因进行文献检索，选出我们感兴趣的基因。高转移潜能细胞株L9981抑癌基因*CDKN2A*（*p16*）、*RUNX3*、*HOXA5*、*PPP2CA*、*APC2*等呈高甲基化，而癌基因*Bcl-2*、*BMI1*呈低甲基化。

**2:** 甲基化差异基因分类 The gene ontology of the hypermethylated and hypomethylated genes in L9981

GO term	Hypermethlated gene (*n*)	Hypomethlated gene (*n*)
Cellular process	531	495
Metabolic process	388	338
Gene expression	189	124
Cell communication	176	191
Signal transduction	153	172
Localization	137	133
Cell adhesion	36	40
Cell-cell signaling	31	32
Cell motility	28	26
Cell migration	23	17
Second-messenger-mediated signaling	23	5
Cell-cell adhesion	14	11
Angiogenesis	12	5
Cell-matrix adhesion	7	5
Cell-substrate adhesion	7	5

表 2所示，大部分甲基化差异基因参与了信号通路的传导，经KEGG进行信号途径的分析，结果发现这些信号途径主要包括：细胞粘附因子、MAPK信号通路、WNT信号通路、TGF-β信号通路等。细胞粘附因子在L9981中主要呈低甲基化，而涉及MAPK、WNT、Notch、Hedgehog通路的基因在L9981中主要呈现高甲基化（表 3）。

**3:** 高转移大细胞肺癌细胞株L9981中甲基化差异基因参与的信号传导通路 The signal transduction of the hypermethylated and hypomethylated genes in L9981 lung cancer cell line

		Hypermethylation		Hypomethylation
Adherent junction	1	*PVRL3*	1	*LMO7*
Calcium signaling pathway	1	*SLC8A2*	2	*CACNA1H, RYR2*
Cell communication	2	*GJB1, LMNB2*		
Cell adhesion molecules (CAMs)	2	*CDH4, CNTNAP2*	8	*CDH2, ICOSLG, MAG, MPZL1, NLGN1, NRCAM, NRXN2, SDC2*
ECM-receptor interaction	3	*COL2A1, COL6A2, TNXB*	2	*COL11A2, TNN*
Focal adhesion	1	*KDR*	2	*COMP, FLNC*
Hedgehog signaling pathway	3	*GAS1, LRP2, ZIC2*		
Insulin signaling pathway	5	*FLOT1, PRKAR1B, RPS6, SKIP, SREBF1*	6	*FBP2, KIAA1303, PFKL, PRKAR1B, PRKAR2B, SKIP*
Jak-STAT signaling path way	1	*IL13RA1*	2	*CISH, CLCF1*
MAPK signaling pathway	3	*DUSP14, JUND, MAPK8IP3*	3	*ATF2, CACNG6, MAPK8IP3*
Notch signaling pathway	3	*JAG2, NCOR2, NOTCH4*		
PPAR signaling pathway	1	*UBC*		
TGF-*β* signaling pathway	3	*ACVRL1, LTBP1, PITX2*	4	*BMPR1A, BMPR2, SMURF1, TFDP1*
Wnt signaling pathway	3	*SFRP2, SFRP4, SOX17*	2	*LRP5, TBL1X*
p53 signaling pathway	2	*CCNB3, IGFBP3*	2	*BBC3, TP53I3*
Toll-like receptor signaling pathway			1	*IRF5*
VEGF signaling pathway			1	*MAPKAPK3*

## 3 讨论

异常甲基化是肿瘤的发生发展的重要原因，主要表现在抑癌基因的高甲基化及全基因组、癌基因的低甲基化^[13, 14]^。我们的研究发现在L9981细胞株中有1 552个高甲基化DNA片断，涉及735个基因，其中包括656个已知基因及79个未知基因。低甲基化DNA片段1 787个，涉及809个基因，其中698个已知基因片段及809个基因，其中698个已知基因片段及111个未知基因。通过DAVID对这些已知基因进行分析，发现它们主要涉及细胞生物进程及其调节、基因表达、信号传导、细胞通讯、细胞运动、细胞粘附及血管生成等，与肿瘤转移关系密切。其中抑癌基因*CDKN2A*（*p16*）、*RUNX3*、*HOXA5*、*PPP2CA*、*APC2*在L9981中为高甲基化，提示他们可能在L9981中表达缺失或下降。而癌基因*Bcl-2*、*BMI1*则在L9981中呈低甲基化，提示他们可能在L9981中过表达。因此我们推测，抑癌基因的高甲基化及癌基因的低甲基化可能是L9981具有更强侵袭及克隆能力的原因。并推测，在L9981中呈高甲基化的基因功能未明或未知基因可能为潜在的肿瘤抑制基因或肿瘤转移抑制基因，但尚需进一步证实。

信号传导通路的激活与肿瘤的发生发展密切相关^[15]^。通过GO分类我们发现两株细胞间大部分的差异甲基化基因参与了信号传导通路，其中与信号通路有关的高甲基化的基因为153个，低甲基化的基因为172个。将这些基因上传至KEGG进行分析发现这些基因涉及细胞粘附因子、MAPK信号通路、WNT信号通路、TGF-β信号通路、p53信号通路。在L9981中，大部分通路的负向调节基因呈现高甲基化，而正向调节基因呈低甲基化。其中WNT通路中负向调节基因*SFRP2*、*SFRP4*、*SOX17*及*RUNX3*、*APC2*、*PPP2CA*等在高转移潜能的细胞株L9981中表现为高甲基化，而正向调节基因*LRP5*、*TBL1X*则为低甲基化。通过GO分类及MILANO文献检索，我们发现在L9981中MAPK信号通路、TGF-β信号通路、p53信号通路均存在负向调节基因的高甲基化及正向调节基因的低甲基化，而Hedgehog及Notch信号通路主要表现为负向调节基因的高甲基化。同时值得一提的是，参与细胞粘附分子通路的基因则以低甲基化为主，其中*CDH2*、*ICOSLG*、*MAG*、*MPZL1*、*NLGN1*、*NRCAM*、*NRXN2*、*SDC2*在L9981细胞株中表现为低甲基化，而*CDH4*、*CNTNAP2*表现为高甲基化，这些基因参与上皮间质转化（epithelial-mesenchymal transitions, EMT）过程^[16, 17]^。而后者为肿瘤浸润转移的重要环节。甲基化在EMT中扮演的作用尚需进一步研究。

肿瘤的侵袭和转移是多因素、多步骤共同作用的结果，DNA甲基化是其中一个因素。运用高通量的基因芯片，我们发现在不同转移潜能的人大细胞肺癌株中甲基化差异基因涉及细胞生物进程及其调节、基因表达、信号传导、细胞通讯、细胞运动、细胞粘附及血管生成等。同时我们注意到在L9981中信号通路负向调节基因的高甲基化及正向调节基因的低甲基化可能促使信号传导通路的开放而促进转移。由于芯片信息量大且受方法学限制，我们尚需对结果进行进一步的挖掘及验证。

## References

[1] Zhou QH (2009). Progress in early diagnosis and screening of lung cancer. China Cancer.

[2] Zhou QH, Wang YP, Che GW (2003). Establishment and their biological characteristics of clonal cell subpopulations (NL9980 and L9981) from a human lung large cell carcinoma cell line (WCQH29801). Chin J Lung Cancer.

[3] Che GW, Zhou QH, Liu LX (2004). *nm23-H_1* gene deletion of cell line screened and identified from human lung cancer lines. Life Sci Res.

[4] Che GW, Zhou QH, Zhu W (2005). Moleculor mechanism of reversing metastatic phenotype in human high-metastatic large cell lung cancer cell line L9981 by nm23-H1. Ai Zheng.

[5] Che GW, Zhou QH, Qin Y (2006). Analysis of the gene expression change in human high-metastatic large cell lung cancer cell line L9981 by microarray before and after transfection with *nm23-H1* gene. Life Science Res.

[6] Woffe AP, Matzke MA (1999). Epigenetics: regulation through repression. Science.

[7] Anisowicz A, Huang H, Braunschweiger KI (2008). A high-throughput and sensitive method to measure global DNA methylation: application in lung cancer. BMC Cancer.

[8] Zhang X, Yazaki J, Sundaresan A (2006). Genome-wide high-resolution mapping and functional analysis of DNA methylation in arabidopsis. Cell.

[9] Rauch T, Wang Z, Zhang X (2007). Homeobox gene methylation in lung cancer studied by genome-wide analysis with a microarray-based methylated CpG island recovery assay. Proc Natl Acad Sci USA.

[10] Fan SC, Zhang XG (2009). Progress of bioinformation in DNA methylation. Prog Biochem Biophys.

[11] Santourlidis S, Florl A, Ackermann R (1999). High frequency of alterations in DNA methylation in adenocarcinoma of the prostate. Prostate.

[12] Wu B, Shen ZY (2006). Data preprocessing of microarray gene expression profile. Chin J Biochem Mol Biol.

[13] SB Baylin, JE Ohm (2006). Epigenetic gene silencing in cancer - a mechanism for early oncogenic pathway addiction?. Nat Rev Caner.

[14] Jaenisch R, Bird A (2003). Epigenetic regulation of gene expression: how the genome integrates intrinsic and environmental signals. Nat Rev Genet.

[15] Licchesi JD, Westra WH, Hooker CM (2008). Epigenetic alteration of Wnt pathway antagonists in progressive glandular neoplasia of the lung. Carcinogenesis.

[16] Miura N, Yano T, Shoji F (2009). Clinicopathological significance of Sip1-associated epithelial mesenchymal transition in non-small cell lung cancer progression. Anticancer Res.

[17] Grinberg-Rashi H, Ofek E, Perelman M (2009). The expression of three genes in primary non-small cell lung cancer is associated with metastatic spread to the brain. Clin Cancer Res.

